# 5-Chloro-1-(4-meth­oxy­benz­yl)indoline-2,3-dione

**DOI:** 10.1107/S1600536810052876

**Published:** 2011-01-08

**Authors:** Weiyao Wu, Tingting Zheng, Shengli Cao, Zhichang Xiao

**Affiliations:** a Department of Chemistry, Capital Normal University, Beijing 100048, People’s Republic of China

## Abstract

In the title compound, C_16_H_12_ClNO_3_, an arm-like 4-meth­oxy­benzene links to 5-chloro­indoline-2,3-dione through a methyl­ene group, with a dihedral angle between the mean planes of the benzene ring and the indole moiety of 88.44 (8)°. In the crystal, weak inter­molecular C—H⋯O and π–π stacking inter­actions [centroid–centroid distance = 3.383 (3) Å] link the mol­ecules together to form a three-dimensional framework.

## Related literature

For the anti­tumor activity of *N*-benzyl isatin analogs, see: Vine *et al.* (2007 ▶); Matesic *et al.* (2008 ▶); Penthala *et al.* (2010 ▶). For the preparation of the title compound, see: Itoh *et al.* (2009 ▶).
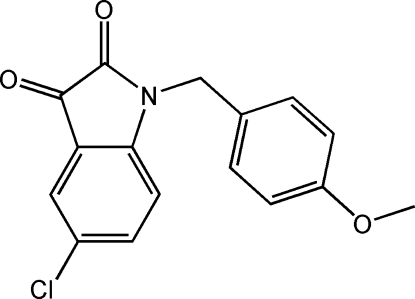


## Experimental

### 

#### Crystal data

C_16_H_12_ClNO_3_
*M* *_r_* = 301.72
Orthorhombic, 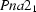

*a* = 7.5318 (17) Å
*b* = 16.587 (4) Å
*c* = 11.220 (3) Å
*V* = 1401.7 (6) Å^3^
*Z* = 4
Mo *K*α radiation
μ = 0.28 mm^−1^
*T* = 296 K
0.30 × 0.22 × 0.10 mm

#### Data collection

Bruker APEXII CCD area-detector diffractometer
7665 measured reflections
3259 independent reflections
2113 reflections with *I* > 2σ(*I*)
*R*_int_ = 0.029

#### Refinement

*R*[*F* ^2^ > 2σ(*F* ^2^)] = 0.040
*wR*(*F* ^2^) = 0.091
*S* = 1.01
3259 reflections
190 parameters
1 restraint
H-atom parameters constrained
Δρ_max_ = 0.11 e Å^−3^
Δρ_min_ = −0.14 e Å^−3^
Absolute structure: Flack (1983 ▶), 1514 Friedel pairs
Flack parameter: −0.01 (7)

### 

Data collection: *APEX2* (Bruker, 2007 ▶); cell refinement: *APEX2* and *SAINT* (Bruker, 2007 ▶); data reduction: *SAINT*; program(s) used to solve structure: *SHELXS97* (Sheldrick, 2008 ▶); program(s) used to refine structure: *SHELXL97* (Sheldrick, 2008 ▶); molecular graphics: *SHELXTL* (Sheldrick, 2008 ▶); software used to prepare material for publication: *SHELXTL* and *PLATON* (Spek, 2009 ▶).

## Supplementary Material

Crystal structure: contains datablocks I, global. DOI: 10.1107/S1600536810052876/zq2080sup1.cif

Structure factors: contains datablocks I. DOI: 10.1107/S1600536810052876/zq2080Isup2.hkl

Additional supplementary materials:  crystallographic information; 3D view; checkCIF report

## Figures and Tables

**Table 1:** Hydrogen-bond geometry (Å, °)

*D*—H⋯*A*	*D*—H	H⋯*A*	*D*⋯*A*	*D*—H⋯*A*
C16—H16*C*⋯O1^i^	0.96	2.63	3.537 (4)	159
C1—H1*A*⋯O2^ii^	0.93	2.58	3.391 (3)	146

Symmetry codes: (i) 
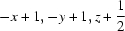
; (ii) 
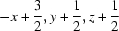
.

**Fig. 1.**
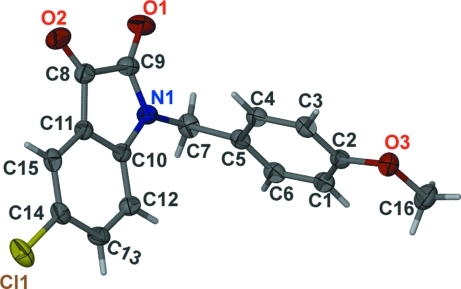
The title molecule with the atomic numbering scheme. The displacement ellipsoids are shown at the 30% probability level, while the hydrogen atoms are shown as rods of arbitrary radius.

**Fig. 2.**
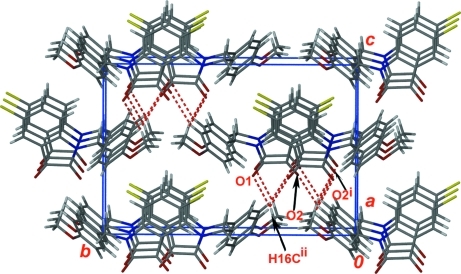
View down the a axis of the packing structure of the title compound. The red dashed lines indicate the intermolecular C—H···O interactions, while the π–π stacking interactions are omitted for clarity. Symmetry codes: (i) x + 1/2, -y + 1/2, z; (ii) -x + 1, -y + 1, z - 1/2.

## supplementary crystallographic information

### Comment

It has been reported that the introduction of a benzyl or naphthylmethyl group into the N1 position of isatin can significantly increase its cytotoxicity against a wide range of human tumor cell lines (Vine *et al.*, 2007; Matesic *et al.*, 2008; Penthala *et al.*, 2010). The studies on the structure-activity relationship of these derivatives also revealed that the 4-methoxybenzyl is one of the most favorable groups for the enhancement of their relative cytotoxicity (Vine *et al.*, 2007). To explore these isatin-based antitumor agents, we synthesized the title compound 5-chloro-1-(4-methoxybenzyl)-indoline-2,3-dione. Herein, we report the structure of the title compound.

In the title compound, C_16_H_12_ClNO_3_, the indoline and methoxybenzene moieties are linked by a methylene group with a C5—C7(methylene)-N1 angle of 113.86 (2)° (Fig. 1). The mean planes of the benzene ring and of the indole-2,3-dione exhibit a dihedral angle of 88.44 (8)°. The molecules stake along the *a* axis and interconnect through π–π interaction between the adjacent indole-2,3-dione moieties, forming a chain structure, as shown in Fig. 2. The distance between the two planes is 3.383 (3) Å. The parallel chains with the stacking molecules are further interconnected through two types of C—H···O(=C) ineractions: C—H(methyl)···O and C—H(benzene)···O (Table 1). The D···A distances vary from 3.391 (3) to 3.537 (4) Å, while the D—H···A angles lie within the 146–159° range. By these cooperative weak intermolecular interactions, a three-dimensional framework is constructed (Fig. 2).

### Experimental

To an ice-bath cooled solution of 5-chloro-indoline-2,3-dione (0.36 g, 2 mmol) in *N,N*-dimethylformamide (20 ml) was added potassium carbonate (0.33 g, 2.4 mmol) and potassium iodide (0.07 g, 0.4 mmol) followed by 4-methoxybenzyl chloride (0.32 ml, 2.2 mmol). The reaction mixture was stirred at 110 °C for 3 h. After cooling to room temperature, the reaction mixture was poured into ice water (80 ml). The resulting precipitate was separated by filtration and purified by column chromatography on silica gel with dichloromethane as an eluent to give the title compound (*R_f_* = 0.81, dichloromethane; m.p. 152–153 °C; yield 72%). The red crystals of the title compound were obtained by slow evaporation from the solution of dichloromethane/ethanol 8:2 (*v*/*v*) at room temperature.

### Refinement

All H atoms were discernible in the difference electron density maps. Nevertheless, the hydrogen atoms were placed into idealized positions and allowed to ride on the carrier atoms, with C—H = 0.93 and 0.97 Å [*U*_iso_(H) = 1.2*U*_eq_(C)] for aromatic and methylene H atoms, respectively, and with C—H = 0.96 Å [*U*_iso_(H) = 1.5*U*_eq_(C)] for methyl H atoms. The Flack parameter is -0.01 (7) in the non-centrosymmetric refinement (1514 Friedel pairs).

### Crystal data

**Table:**

C_16_H_12_ClNO_3_	*Z* = 4
*M**_r_* = 301.72	*F*(000) = 624
Orthorhombic, *P**n**a*2_1_	*D*_x_ = 1.430 Mg m^−^^3^*D*_m_ = 1.430 Mg m^−^^3^*D*_m_ measured by not measured
Hall symbol: P 2c -2n	Mo *K*α radiation, λ = 0.71073 Å
*a* = 7.5318 (17) Å	µ = 0.28 mm^−^^1^
*b* = 16.587 (4) Å	*T* = 296 K
*c* = 11.220 (3) Å	Block, red
*V* = 1401.7 (6) Å^3^	0.30 × 0.22 × 0.10 mm

### Data collection

**Table:**

Bruker APEXII CCD area-detector diffractometer	2113 reflections with *I* > 2σ(*I*)
Radiation source: fine-focus sealed tube	*R*_int_ = 0.029
graphite	θ_max_ = 27.9°, θ_min_ = 2.2°
φ and ω scans	*h* = −9→6
7665 measured reflections	*k* = −21→21
3259 independent reflections	*l* = −14→14

### Refinement

**Table:**

Refinement on *F*^2^	Secondary atom site location: difference Fourier map
Least-squares matrix: full	Hydrogen site location: inferred from neighbouring sites
*R*[*F*^2^ > 2σ(*F*^2^)] = 0.040	H-atom parameters constrained
*wR*(*F*^2^) = 0.091	*w* = 1/[σ^2^(*F*_o_^2^) + (0.0356*P*)^2^ + 0.0529*P*] where *P* = (*F*_o_^2^ + 2*F*_c_^2^)/3
*S* = 1.01	(Δ/σ)_max_ < 0.001
3259 reflections	Δρ_max_ = 0.11 e Å^−^^3^
190 parameters	Δρ_min_ = −0.14 e Å^−^^3^
1 restraint	Absolute structure: Flack (1983), 1514 Friedel pairs
Primary atom site location: structure-invariant direct methods	Flack parameter: −0.01 (7)

### Special details

**Table:**

Geometry. All e.s.d.'s (except the e.s.d. in the dihedral angle between two l.s. planes) are estimated using the full covariance matrix. The cell e.s.d.'s are taken into account individually in the estimation of e.s.d.'s in distances, angles and torsion angles; correlations between e.s.d.'s in cell parameters are only used when they are defined by crystal symmetry. An approximate (isotropic) treatment of cell e.s.d.'s is used for estimating e.s.d.'s involving l.s. planes.
Refinement. Refinement of *F*^2^ against ALL reflections. The weighted *R*-factor *wR* and goodness of fit *S* are based on *F*^2^, conventional *R*-factors *R* are based on *F*, with *F* set to zero for negative *F*^2^. The threshold expression of *F*^2^ > σ(*F*^2^) is used only for calculating *R*-factors(gt) *etc*. and is not relevant to the choice of reflections for refinement. *R*-factors based on *F*^2^ are statistically about twice as large as those based on *F*, and *R*- factors based on ALL data will be even larger.

### Fractional atomic coordinates and isotropic or equivalent isotropic displacement parameters (Å^2^)

**Table:**

	*x*	*y*	*z*	*U*_iso_*/*U*_eq_	
Cl1	0.68342 (11)	0.09999 (5)	0.75885 (8)	0.0978 (3)	
O1	1.0165 (3)	0.41051 (12)	0.32537 (18)	0.0858 (6)	
O2	1.0012 (3)	0.23283 (12)	0.33272 (17)	0.0861 (6)	
O3	0.1766 (2)	0.61539 (11)	0.48186 (16)	0.0694 (5)	
N1	0.8772 (3)	0.39659 (11)	0.50677 (17)	0.0571 (5)	
C1	0.4538 (4)	0.61020 (13)	0.5928 (2)	0.0611 (7)	
H1A	0.4197	0.6518	0.6435	0.073*	
C2	0.3403 (3)	0.58404 (13)	0.5057 (2)	0.0542 (6)	
C3	0.3907 (3)	0.52070 (13)	0.4320 (2)	0.0573 (6)	
H3A	0.3127	0.5014	0.3745	0.069*	
C4	0.5565 (3)	0.48649 (13)	0.4443 (2)	0.0545 (6)	
H4A	0.5903	0.4448	0.3937	0.065*	
C5	0.6734 (3)	0.51321 (13)	0.5309 (2)	0.0528 (6)	
C6	0.6183 (4)	0.57500 (13)	0.6055 (2)	0.0600 (6)	
H6A	0.6940	0.5930	0.6653	0.072*	
C7	0.8599 (3)	0.48135 (14)	0.5399 (2)	0.0656 (7)	
H7A	0.9362	0.5133	0.4887	0.079*	
H7B	0.9012	0.4881	0.6212	0.079*	
C8	0.9424 (3)	0.27675 (16)	0.4091 (2)	0.0619 (6)	
C9	0.9526 (3)	0.37002 (16)	0.4045 (2)	0.0624 (6)	
C10	0.8176 (3)	0.33139 (13)	0.5770 (2)	0.0489 (5)	
C11	0.8551 (3)	0.25832 (13)	0.52206 (19)	0.0515 (5)	
C12	0.7381 (3)	0.33395 (14)	0.6865 (2)	0.0585 (6)	
H12A	0.7128	0.3829	0.7232	0.070*	
C13	0.6964 (3)	0.26183 (16)	0.7410 (2)	0.0619 (6)	
H13A	0.6416	0.2621	0.8153	0.074*	
C14	0.7354 (3)	0.18880 (15)	0.6861 (2)	0.0611 (6)	
C15	0.8143 (3)	0.18620 (13)	0.5764 (2)	0.0588 (6)	
H15A	0.8396	0.1373	0.5397	0.071*	
C16	0.1141 (4)	0.67967 (17)	0.5534 (3)	0.0917 (10)	
H16A	−0.0019	0.6956	0.5268	0.138*	
H16B	0.1939	0.7246	0.5470	0.138*	
H16C	0.1078	0.6625	0.6350	0.138*	

### Atomic displacement parameters (Å^2^)

**Table:**

	*U*^11^	*U*^22^	*U*^33^	*U*^12^	*U*^13^	*U*^23^
Cl1	0.1025 (6)	0.0832 (5)	0.1078 (6)	−0.0071 (4)	−0.0163 (5)	0.0446 (5)
O1	0.0912 (14)	0.0996 (15)	0.0667 (12)	−0.0161 (12)	0.0144 (11)	0.0145 (11)
O2	0.0987 (15)	0.0962 (14)	0.0634 (11)	0.0152 (12)	0.0085 (11)	−0.0211 (11)
O3	0.0674 (12)	0.0639 (11)	0.0768 (12)	0.0064 (9)	−0.0015 (9)	−0.0028 (9)
N1	0.0626 (12)	0.0543 (12)	0.0542 (12)	−0.0024 (9)	0.0045 (10)	−0.0012 (9)
C1	0.0826 (19)	0.0469 (12)	0.0538 (15)	0.0005 (13)	0.0015 (13)	−0.0052 (11)
C2	0.0622 (15)	0.0478 (13)	0.0528 (14)	−0.0045 (11)	0.0026 (13)	0.0042 (10)
C3	0.0680 (16)	0.0484 (13)	0.0555 (15)	−0.0070 (12)	−0.0092 (13)	−0.0014 (11)
C4	0.0656 (16)	0.0463 (13)	0.0516 (14)	−0.0069 (12)	0.0003 (12)	−0.0040 (10)
C5	0.0626 (15)	0.0420 (11)	0.0536 (13)	−0.0073 (11)	−0.0012 (12)	0.0035 (10)
C6	0.0775 (18)	0.0508 (13)	0.0515 (14)	−0.0073 (13)	−0.0070 (12)	−0.0052 (11)
C7	0.0719 (17)	0.0473 (13)	0.0775 (18)	−0.0101 (12)	−0.0107 (14)	−0.0034 (12)
C8	0.0628 (16)	0.0724 (16)	0.0504 (14)	0.0086 (13)	−0.0054 (12)	−0.0097 (13)
C9	0.0605 (16)	0.0754 (16)	0.0513 (14)	−0.0047 (13)	0.0019 (13)	0.0033 (14)
C10	0.0485 (12)	0.0515 (12)	0.0468 (12)	0.0001 (11)	−0.0045 (10)	−0.0013 (11)
C11	0.0510 (12)	0.0551 (13)	0.0482 (13)	0.0033 (10)	−0.0085 (11)	−0.0051 (11)
C12	0.0649 (15)	0.0583 (14)	0.0522 (14)	−0.0016 (12)	0.0009 (12)	−0.0101 (11)
C13	0.0601 (15)	0.0795 (17)	0.0460 (13)	−0.0036 (13)	−0.0033 (12)	0.0020 (13)
C14	0.0565 (15)	0.0637 (15)	0.0630 (16)	−0.0028 (12)	−0.0136 (13)	0.0138 (13)
C15	0.0588 (15)	0.0506 (13)	0.0669 (16)	0.0049 (11)	−0.0166 (13)	−0.0035 (12)
C16	0.087 (2)	0.088 (2)	0.100 (2)	0.0183 (17)	0.0142 (18)	−0.0122 (18)

### Geometric parameters (Å, °)

**Table:**

Cl1—C14	1.729 (2)	C6—H6A	0.9300
O1—C9	1.213 (3)	C7—H7A	0.9700
O2—C8	1.208 (3)	C7—H7B	0.9700
O3—C2	1.364 (3)	C8—C11	1.461 (3)
O3—C16	1.415 (3)	C8—C9	1.550 (4)
N1—C9	1.354 (3)	C10—C12	1.367 (3)
N1—C10	1.412 (3)	C10—C11	1.389 (3)
N1—C7	1.460 (3)	C11—C15	1.378 (3)
C1—C2	1.369 (3)	C12—C13	1.380 (3)
C1—C6	1.378 (3)	C12—H12A	0.9300
C1—H1A	0.9300	C13—C14	1.391 (3)
C2—C3	1.390 (3)	C13—H13A	0.9300
C3—C4	1.379 (3)	C14—C15	1.367 (4)
C3—H3A	0.9300	C15—H15A	0.9300
C4—C5	1.384 (3)	C16—H16A	0.9600
C4—H4A	0.9300	C16—H16B	0.9600
C5—C6	1.386 (3)	C16—H16C	0.9600
C5—C7	1.504 (3)		
			
C2—O3—C16	118.5 (2)	O2—C8—C9	124.1 (2)
C9—N1—C10	110.92 (18)	C11—C8—C9	105.0 (2)
C9—N1—C7	124.5 (2)	O1—C9—N1	127.4 (2)
C10—N1—C7	124.6 (2)	O1—C9—C8	126.6 (2)
C2—C1—C6	120.1 (2)	N1—C9—C8	106.1 (2)
C2—C1—H1A	120.0	C12—C10—C11	121.0 (2)
C6—C1—H1A	120.0	C12—C10—N1	128.1 (2)
O3—C2—C1	125.7 (2)	C11—C10—N1	110.85 (19)
O3—C2—C3	114.8 (2)	C15—C11—C10	121.0 (2)
C1—C2—C3	119.6 (2)	C15—C11—C8	131.8 (2)
C4—C3—C2	119.9 (2)	C10—C11—C8	107.1 (2)
C4—C3—H3A	120.0	C10—C12—C13	118.1 (2)
C2—C3—H3A	120.0	C10—C12—H12A	121.0
C3—C4—C5	121.0 (2)	C13—C12—H12A	121.0
C3—C4—H4A	119.5	C12—C13—C14	120.7 (2)
C5—C4—H4A	119.5	C12—C13—H13A	119.6
C4—C5—C6	118.1 (2)	C14—C13—H13A	119.6
C4—C5—C7	121.9 (2)	C15—C14—C13	121.2 (2)
C6—C5—C7	119.9 (2)	C15—C14—Cl1	119.8 (2)
C1—C6—C5	121.3 (2)	C13—C14—Cl1	119.0 (2)
C1—C6—H6A	119.3	C14—C15—C11	117.9 (2)
C5—C6—H6A	119.3	C14—C15—H15A	121.0
N1—C7—C5	113.85 (19)	C11—C15—H15A	121.0
N1—C7—H7A	108.8	O3—C16—H16A	109.5
C5—C7—H7A	108.8	O3—C16—H16B	109.5
N1—C7—H7B	108.8	H16A—C16—H16B	109.5
C5—C7—H7B	108.8	O3—C16—H16C	109.5
H7A—C7—H7B	107.7	H16A—C16—H16C	109.5
O2—C8—C11	130.8 (2)	H16B—C16—H16C	109.5
			
C16—O3—C2—C1	−1.2 (3)	C11—C8—C9—N1	0.1 (3)
C16—O3—C2—C3	179.4 (2)	C9—N1—C10—C12	178.7 (2)
C6—C1—C2—O3	−178.0 (2)	C7—N1—C10—C12	−1.2 (4)
C6—C1—C2—C3	1.4 (3)	C9—N1—C10—C11	0.7 (3)
O3—C2—C3—C4	177.2 (2)	C7—N1—C10—C11	−179.2 (2)
C1—C2—C3—C4	−2.2 (3)	C12—C10—C11—C15	−0.4 (3)
C2—C3—C4—C5	1.3 (3)	N1—C10—C11—C15	177.8 (2)
C3—C4—C5—C6	0.5 (3)	C12—C10—C11—C8	−178.8 (2)
C3—C4—C5—C7	−175.1 (2)	N1—C10—C11—C8	−0.6 (2)
C2—C1—C6—C5	0.4 (3)	O2—C8—C11—C15	0.5 (5)
C4—C5—C6—C1	−1.3 (3)	C9—C8—C11—C15	−177.9 (2)
C7—C5—C6—C1	174.4 (2)	O2—C8—C11—C10	178.7 (3)
C9—N1—C7—C5	107.0 (3)	C9—C8—C11—C10	0.3 (2)
C10—N1—C7—C5	−73.1 (3)	C11—C10—C12—C13	0.1 (3)
C4—C5—C7—N1	−33.9 (3)	N1—C10—C12—C13	−177.7 (2)
C6—C5—C7—N1	150.6 (2)	C10—C12—C13—C14	0.4 (3)
C10—N1—C9—O1	179.9 (2)	C12—C13—C14—C15	−0.6 (4)
C7—N1—C9—O1	−0.1 (4)	C12—C13—C14—Cl1	179.08 (19)
C10—N1—C9—C8	−0.5 (3)	C13—C14—C15—C11	0.4 (3)
C7—N1—C9—C8	179.5 (2)	Cl1—C14—C15—C11	−179.33 (18)
O2—C8—C9—O1	1.2 (4)	C10—C11—C15—C14	0.1 (3)
C11—C8—C9—O1	179.7 (2)	C8—C11—C15—C14	178.0 (2)
O2—C8—C9—N1	−178.4 (2)		

### Hydrogen-bond geometry (Å, °)

**Table:**

*D*—H···*A*	*D*—H	H···*A*	*D*···*A*	*D*—H···*A*
C16—H16C···O1^i^	0.96	2.63	3.537 (4)	159
C1—H1A···O2^ii^	0.93	2.58	3.391 (3)	146

Symmetry codes: (i) −*x*+1, −*y*+1, *z*+1/2; (ii) −*x*+3/2, *y*+1/2, *z*+1/2.
